# Locating the quantum critical point of the Bose-Hubbard model through singularities of simple observables

**DOI:** 10.1038/srep38340

**Published:** 2016-12-02

**Authors:** Mateusz Łącki, Bogdan Damski, Jakub Zakrzewski

**Affiliations:** 1 Instytut Fizyki imienia Mariana Smoluchowskiego, Uniwersytet Jagielloński, ulica Łojasiewicza 11, 30-348 Kraków, Poland; 2 Institute for Quantum Optics and Quantum Information of the Austrian Academy of Sciences, A-6020 Innsbruck, Austria; 3 Institute for Theoretical Physics, University of Innsbruck, A-6020 Innsbruck, Austria

## Abstract

We show that the critical point of the two-dimensional Bose-Hubbard model can be easily found through studies of either on-site atom number fluctuations or the nearest-neighbor two-point correlation function (the expectation value of the tunnelling operator). Our strategy to locate the critical point is based on the observation that the derivatives of these observables with respect to the parameter that drives the superfluid-Mott insulator transition are singular at the critical point in the thermodynamic limit. Performing the quantum Monte Carlo simulations of the two-dimensional Bose-Hubbard model, we show that this technique leads to the accurate determination of the position of its critical point. Our results can be easily extended to the three-dimensional Bose-Hubbard model and different Hubbard-like models. They provide a simple experimentally-relevant way of locating critical points in various cold atomic lattice systems.

The amazing recent progress in cold atom manipulations allows for experimental studies of strongly correlated bosonic systems placed in lattices of various dimensions and shapes^1^,^2^,^3^. The basic physics of such systems is captured by the Bose-Hubbard model^1^,^2^,^3^,^4^,^5^,^6^, whose Hamiltonian has the following deceptively simple form


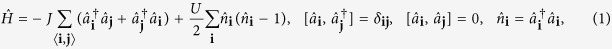


where 〈**i**, **j**〉 stands for nearest-neighbor lattice sites **i** and **j**, while 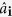 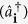 annihilates (creates) an atom in the **i**-th lattice site. The first term in this Hamiltonian describes nearest-neighbor tunnelling, while the second one describes repulsive on-site interactions. Thus, the former term promotes spreading of atoms across the lattice, which leads to on-site atom number fluctuations that are being suppressed by the latter term.

The competition between the tunnelling and interactions leads to the quantum phase transition when the average number of atoms per lattice site (the filling factor) is integer^4^. The system is in the Mott insulator phase when *J*/*U* < (*J*/*U*)_*c*_ and it is in the superfluid phase when *J*/*U* > (*J*/*U*)_*c*_.

The location of the critical point depends on the dimensionality of the system and the filling factor. We are primarily interested here in the two-dimensional (2D) Bose-Hubbard model at unit filling factor. Such a model can be emulated in a cold atom cloud placed in the optical lattice generated by three standing laser beams producing the periodic potential


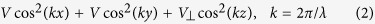


for atoms (see refs 7, 8, 9, 10, 11, 12 for experimental studies of the 2D Bose-Hubbard model). Above *λ* is the wavelength of the laser beams, *V* is the height of the optical potential in the *x*–*y* plane where we study the 2D Bose-Hubbard model, and *V*_⊥_ is the height of the lattice potential confining the atoms to this plane 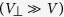. Additionally, we assume that atoms are kept in the optical lattice by the optical box trap enabling studies of homogeneous systems (see refs 13 and 14 for experiments on cold atoms in box potentials).

The position of the critical point in the 2D Bose-Hubbard model at unit filling factor has been discussed in numerous theoretical studies and an agreement has been reached that


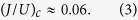


This value was obtained by theoretical studies of the superfluid density^15^, compressibility^16^, structure factor^17^, energy gap^18^,^19^,^20^,^21^, entanglement entropy^22^, fidelity susceptibility^23^, fixed points of the real-space renormalization group flow^24^, and superfluid order parameter^25^,^26^. We will discuss below two more observables that can be used to locate the critical point. They are in our view the most natural experimentally-relevant observables one can think of in the context of the Bose-Hubbard model.

## Results

### Idea

To proceed, it is convenient to introduce the parameter


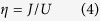


and denote the ground state of the Hamiltonian (1) as |*η*〉.

Our idea is to locate the critical point of the 2D Bose-Hubbard model through the *singularities* of the *derivatives* of either the nearest-neighbor two-point correlation function


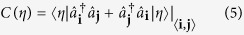


or the variance of on-site atom number operator


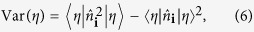


which has not been done before.

More precisely, we propose that for some specific large-enough *r*


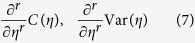


will be divergent at the critical point in the thermodynamically-large system at zero absolute temperature. The singularity of (7) follows from the defining feature of a quantum phase transition: Non-analyticity of the ground-state energy at the quantum critical point (Ch. 1.1 of ref. 27; see also ref. 28 for a pleasant introduction to quantum phase transitions).

We proceed in the standard way to verify this claim. We introduce the ground-state energy 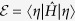, note that 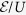 is a function of *η* only, use the Feynman-Hellman theorem


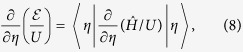


and take into account that


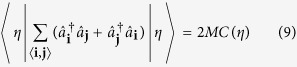


and


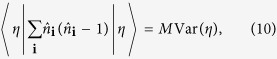


where *M* is the number of lattice sites. The 2D square lattice geometry and unit filling factor are assumed in equations (9) and (10), respectively. The translational invariance of the ground state is also assumed in these equations. Generalization of equations (9) and (10) to other lattice geometries and filling factors is straightforward.

Using equations (8, 9, 10) one finds that


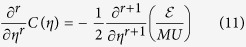


and


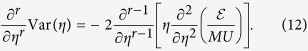


As the quantum critical point is traditionally associated with non-analyticity of the ground-state energy, we assume that the derivatives of the ground-state energy with respect to the parameter driving the transition,


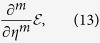


are continuous for *m* = 0, …, *r* and either divergent or discontinuous at the critical point for *m* = *r* + 1.

The question now is which derivative of the ground-state energy should we expect to be divergent? To answer this question, we note that the quantum phase transition of the 2D Bose-Hubbard model lies in the universality class of the classical 3D XY model^4^, whose singular part of the free energy scales with the distance *t* from the critical point as *f*_*s*_(*t*) ~ *t*^2−*α*^, where *α* equals about −0.0136 (see refs 29 and 30). Following the discussion in Ch. 1.7 of ref. 31, we write 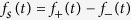, where *f*_+_ (*f*_−_) is the free energy for *t* > 0 (*t* < 0), which has been analytically continued to the complex *t* plane. Non-analyticity at the critical point is then seen by non-zero derivative(s) of *f*_*s*_ at *t* = 0. The third and higher derivatives of *f*_*s*_ are divergent at the critical point of the classical 3D XY model. Going back from the classical 3D XY model to the quantum 2D Bose-Hubbard model, we expect that the third derivative of the ground-state energy will be singular. This translates into the divergent second derivative of both nearest-neighbor correlation function and variance through (11) and (12), respectively. Such singularity of *C*(*η*) and Var(*η*) is expected to develop in the limits of temperature *T* → 0 and the system size *M* → ∞. For finite systems instead of a singularity either an extremum or a kink smoothing out discontinuity should develop near the critical point for small enough temperatures. We will now discuss the observables that we use for finding the critical point.

### Observables

The correlation functions 
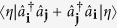
 are arguably the most experimentally accessible correlation functions in cold atom systems. It is so because their Fourier transform provides a quasi-momentum distribution of cold atom clouds, which can be extracted from the time-of-flight images^32^,^33^ (see refs 7, 8, 9 for measurements in the 2D Bose-Hubbard system).

The critical point can be extracted from these correlation functions through the study of their decay with the distance |**i** − **j**| between the lattice sites. They are expected to decay exponentially in the Mott phase and algebraically in the superfluid phase in the thermodynamically-large zero-temperature system. Such a strategy of finding the critical point in the 2D Bose-Hubbard model is problematic because the exponential vs. algebraic transition is expected to happen for large |**i** − **j**| distances. Such distances are hard to deal with in theoretical calculations because the model is not exactly solvable and it does require substantial computational resources to handle moderate lattices sizes. On the cold atom experimental side, one has to face issues with accurate measurement of distant correlation functions.

Therefore, we would like to argue that our approach provides a more practical way of locating the critical point as it is based on the nearest-neighbor two-point correlation function, which among other correlation functions is the easiest to obtain both theoretically and experimentally.

The variance of the on-site atom occupation can be estimated *in*-*situ* thanks to the recent breakthrough in the quantum gas microscopy^34^. This technique allows for the detection of 0, 1, 2, 3 atoms in individual lattice sites. Choosing the sites far away from the borders of the trap, one should be able to minimize the influence of finite-size effects, which should facilitate extraction of the critical point from the experimental data.

The derivatives of the two observables, *C*(*η*) and Var(*η*), are proportional to each other^35^


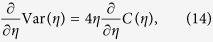


so it suffices to measure either one of them.

Therefore, we would like to stress that the measurements of either *C*(*η*) or Var(*η*) are possible in the current state-of-the-art experimental setups. In fact, the measurements of *C*(*η*) have been possible since the seminal paper of Greiner *et al*.^32^. It is thus a little bit surprising that nobody has studied the derivatives of at least *C*(*η*) to obtain unambiguous signatures of a superfluid-Mott insulator quantum phase transition. The idea to find the critical point through the expectation values (or thermodynamical averages in classical phase transitions) of different terms of the Hamiltonian is quite natural and has been explored before (see e.g. ref. 31 in the context of classical and ref. 36 in the context of quantum phase transitions). To the best of our knowledge, however, *non*-*analytic* properties of these expectation values have not been explored in the context of cold atomic systems. We fill this gap by presenting the following quantum Monte Carlo simulations that we hope will motivate future experimental efforts.

### Quantum Monte Carlo simulations

We perform quantum Monte Carlo (QMC) simulations of the 2D Bose-Hubbard model (1) imposing periodic boundary conditions on the lattice^37^,^38^. We divide the Hamiltonian by *U*, thereby choosing *U* as the unit of energy, and set *η* as the parameter driving the transition (4).

We compute the variance of the on-site atom occupation for lattice sizes *M* = 10^2^ to 40^2^ and temperatures *k*_*B*_*T*/*U* between 0.005 and 0.08. To estimate what such temperatures correspond to, we assume two plausible experimental setups. Namely, ^23^Na and ^87^Rb atoms placed in the lattice (2) with *λ* = 532 nm and *V*_⊥_ = 30 *E*_*R*_, where the recoil energy *E*_*R*_ = *ħ*^2^*k*^2^/2 *m* with *m* being the mass of the atom. As the s-wave scattering lengths we take 2.8 nm for sodium and 5.3 nm for rubidium. Computing Wannier functions and proceeding in the standard way^39^, we find that the critical point (3) for the sodium (rubidium) system is located at the height *V* of the lattice potential equal to 10.1 *E*_*R*_ (8.0 *E*_*R*_), for which the coefficient *U* equals 0.31 *E*_*R*_ (0.51 *E*_*R*_). Having these coefficients, one finds that *U*/*k*_*B*_ at the critical point equals 461 nK for sodium and 199 nK for rubidium, respectively. Combining these results, we see that the highest temperatures that we consider are 37 nK (16 nK) for the above-proposed sodium (rubidium) setup. Both temperatures are experimentally accessible^9^,^10^,^11^,^12^.

**Figure 1:**
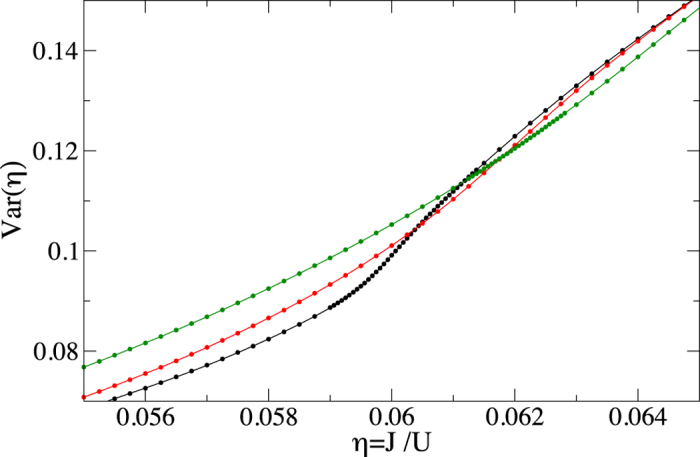
The variance of the on-site number operator. The circles provide QMC data while the solid lines are Padé approximants (15) fitted to the numerics with parameters *m* ≤ 9 and *n* ≤ 6 (the lower the temperature the higher order polynomials are needed to fit the numerics). The curves from bottom to top (in the left part of the plot) correspond to temperatures *k*_*B*_*T*/*U* equal to 0.005 (black), 0.04 (red), 0.06 (green), respectively. The system size is *M* = 40^2^.

**Figure 2:**
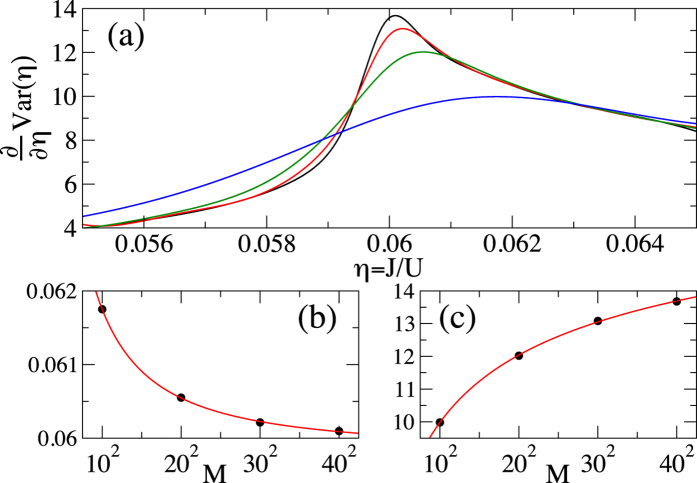
Plot (**a**) First derivative of the variance of the on-site number operator. The curves from top to bottom (around the maximum) correspond to *M* equal to 40^2^ (black), 30^2^ (red), 20^2^ (green), and 10^2^ (blue), respectively. Plot (**b**) The position of the maximum of ∂_*η*_Var. Plot (**c**) ∂_*η*_Var at the maximum. The circles in plots (**b**) and (**c**) show QMC data, while the solid lines provide fits (16) and (17), respectively. All plots are for *k*_*B*_*T*/*U* = 0.01.

**Figure 3:**
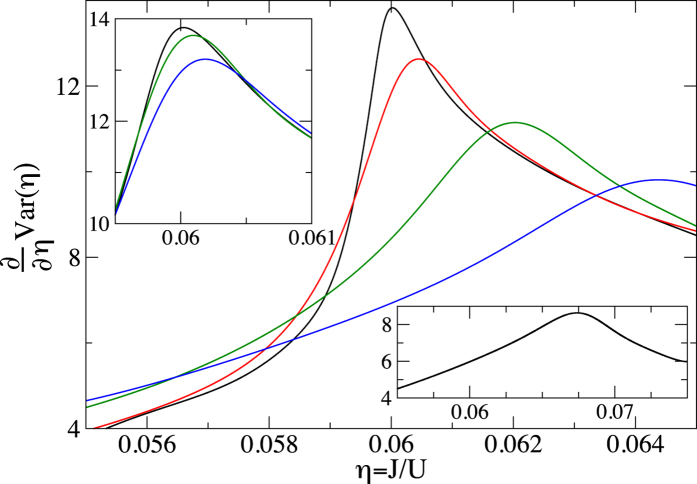
Derivative of the variance of the on-site number operator. The curves from top to bottom in the main plot correspond to temperatures *k*_*B*_*T*/*U* equal to 0.005 (black), 0.02 (red), 0.04 (green), 0.06 (blue), respectively. Upper inset illustrates the convergence of our results towards the *T* = 0 limit. It shows our three lowest-temperature results: from top to bottom *k*_*B*_*T*/*U* equals 0.005 (black), 0.01 (green), and 0.015 (blue), respectively. Lower inset shows the same as the main plot but for *k*_*B*_*T*/*U* = 0.08. The system size is *M* = 40^2^ for all the calculations in this figure.

**Figure 4:**
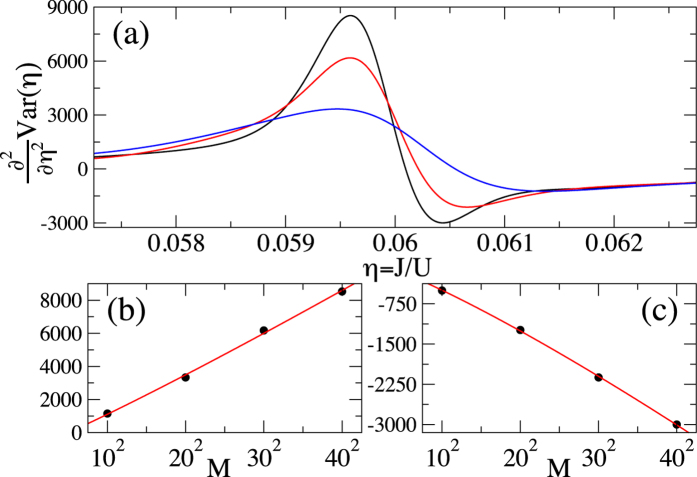
Plot (**a**) Second derivative of the variance of the on-site number operator. The curves from top to bottom (around the maximum) correspond to *M* equal to 40^2^ (black), 30^2^ (red), and 20^2^ (blue), respectively. Plot (**b**) 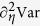 at the maximum. Plot (**c**) 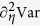 at the minimum. The circles in plots (**b**) and (**c**) show QMC data, while the solid lines provide the fit (17). All plots are for *k*_*B*_*T*/*U* = 0.01.

The results that we obtain are presented in Figs 1, 2, 3 and 4. To be able to accurately extract the derivatives of the variance, we fit Padé approximants


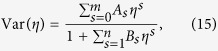


to the QMC numerics (Fig. 1) and then differentiate the resulting curves (Figs 2, 3 and 4). Such a procedure removes the influence of small fluctuations in the QMC calculations on our results. Moreover, it can be straightforwardly applied to experimental data that will be affected in a similar way by the limited accuracy of measurements.

Looking at Fig. 1, we see that for the lowest temperature displayed, *k*_*B*_*T*/*U* = 0.005, there is a steep increase of the variance around the critical point (3). Such an abrupt increase is reminiscent of the behavior of magnetization of the 1D quantum Ising model in the transverse field near the critical point^40^. As temperature rises, the abrupt growth of the variance near the critical point fades away and the variance seems to be featureless, which is illustrated for *k*_*B*_*T*/*U* = 0.04 and 0.06 in Fig. 1. It is thus worth to stress that the position of the critical point is beautifully encoded in all the curves from Fig. 1.

In order to extract it, we compute ∂_*η*_Var, where ∂_*η*_ = ∂/∂*η*, finding that it has a maximum very close to the critical point (Fig. 2). The position of the maximum, *η*_max_(*M*), moves towards the critical point as the system size is increased. To extrapolate it to the thermodynamic limit, we fit


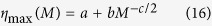


to QMC data for *M* = 10^2^ − 40^2^ and *k*_*B*_*T*/*U* = 0.01 (Fig. 2b; all the fits below are also done for these parameters). We obtain *a* = 0.0598, *b* = 0.0491, *c* = 1.40. It turns out that the value of the parameter *a* = *η*_max_(∞) is the same as the most accurate estimations of the position of the critical point in the 2D Bose-Hubbard model^16^,^20^,^21^.

Furthermore, we observe that if we fix the system size and vary temperature, then the position of the maximum of ∂_*η*_Var approaches the critical point when *T* → 0 (Fig. 3). Moreover, we observe that ∂_*η*_Var at the maximum grows with both the system size (Fig. 2) and the inverse of the temperature (Fig. 3).

Therefore, it is interesting to ask whether the studied maximum is in fact the singularity that is rounded off and shifted away from the critical point by finite-size effects. To investigate it, we fit ∂_*η*_Var(*η*_max_) with


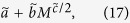


getting 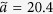, 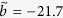, 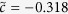 (Fig. 2c). Taking the limit of *M* → ∞, we find that instead of a singularity there is a maximum of ∂_*η*_Var in the thermodynamic limit.

Hunting for a singularity, we compute 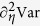 getting the maximum and minimum near the critical point (Fig. 4a). The study of 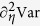 at the extrema through the fit (17) supports the conclusion that there is a singularity appearing in the thermodynamic limit (Fig. 4b and c). Indeed, for maximum (minimum) we get 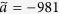, 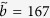, 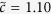 (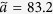, 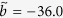, 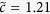). This means that as *M* → ∞, we have 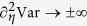 at the extrema. In-between these extrema there is the point where 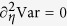, i.e., where the maximum of ∂_*η*_Var is located. Thus, in the thermodynamic limit the divergent discontinuity of 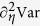 will be located at the same point as the maximum of ∂_*η*_Var (if that wouldn’t be the case, then there would be two points where 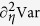 is non-analytic, which would contradict presence of a single critical point in the system). This observation explains why the non-singular in the thermodynamic limit maximum of ∂_*η*_Var encodes the position of the critical point so accurately.

The extrapolated thermodynamic-limit singularity of 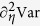 implies the singularity of the third derivative of the ground-state energy (12), which agrees with the above-presented discussion based on the scaling theory of phase transitions. It is worth to stress that it is so because the critical exponent −1 < *α* < 0. Such an exponent can be directly experimentally measured near the lambda transition in liquid ^4^He, which also belongs to the universality class of the classical 3D XY model (see e.g. ref. 30 reporting the outcome of an experiment done in a Space Shuttle to eliminate the influence of gravity on the transition). On the theoretical side, one can obtain this exponent through the hyperscaling relation linking it to more commonly studied critical exponents^41^


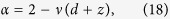


where *ν* is the exponent providing algebraic divergence of the correlation length, *z* is the dynamical exponent relating the excitation gap to the inverse correlation length, and *d* is the dimensionality of the quantum system. In our system *d* = 2 and *z* = 1 and so 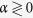 for 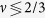. Since the critical exponent *ν* is nearly 2/3 in the 2D Bose-Hubbard model, its accurate determination is needed to find out whether *α* is a little bit smaller or greater than zero. Had the latter possibility been realized, the second derivative of the ground-state energy (the first derivative of either variance or nearest-neighbor correlation function) would have been divergent.

Finally, we mention that the standard expectation coming from the finite-size scaling theory is that thermodynamic-limit singularities are rounded off and shifted away from the critical point by the distance ~*M*^−*ϕ*/*d*^, where *ϕ* is an integer multiple of 1/*ν* (see refs 42 and 43). Fitting the position of the maximum (minimum) of 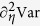 with (16), we obtain *a* = 0.0596, *b* = −0.542, *c* = 2.74 (*a* = 0.0598, *b* = 0.0788, *c* = 1.30). Thus, we see that the fitting parameter *c* roughly matches integer multiples of 1/*ν* ≈ 1.49 in the 2D Bose-Hubbard model^29^,^30^. The same conclusion applies to the finite-size scaling of the position of the maximum of ∂_*η*_Var; see the fitting results right below (16). Simulations of larger system sizes are needed for making conclusive predictions about the relation between *c* and 1/*ν*.

## Discussion

We have shown that derivatives of the nearest-neighbor correlation function and the variance of on-site atom number operator can be used as an efficient and experimentally-relevant probe of the location of the critical point of the 2D Bose-Hubbard model.

Similar calculations can be performed for the 3D Bose-Hubbard model, where *z* = 1 and *ν* = 1/2 (see ref. 4). Using equation (18) one then finds that *α* = 0. Therefore, based on the discussion from the Results section, we expect that the second derivative of the ground-state energy as well as the first derivative of nearest-neighbor correlation function and the variance of the on-site atom number operator will be divergent at the critical point of this model. This conclusion applies to the thermodynamically-large zero-temperature system, while in the finite-size system we expect to find an extremum of these observables near the critical point just as in the 2D Bose-Hubbard model.

Interestingly, in the 1D quantum Ising model in the transverse field, where *z* = *ν* = 1, *α* = 0 as well. In this model a closed-form expression for the ground-state energy is known^40^ and one can check that indeed the second derivative of the ground-state energy is divergent at the critical point of this model.

Furthermore, we would like to mention that it is unclear at the moment whether one can extract the position of the critical point of the 1D Bose-Hubbard model in a similar way. The problem is that such a model undergoes a Berezinskii-Kosterlitz-Thouless (BKT) transition^4^, where the singularities associated with the critical point are not algebraic but exponential in the distance from the critical point^44^. As a result, the above discussion of the singularity of the ground-state energy based on the exponent *α* is not readily applicable as it assumes that the divergence of the correlation length is algebraic ~|*t*|^−*ν*^, where again *t* is the distance from the critical point.

Finally, we would like to mention that the discussion from the Results section can be straightforwardly extended to Hubbard-like models undergoing a regular, i.e. not a BKT-type, transition. Different such models can be studied with cold atoms in optical lattices and one can consider not only bosonic but also fermionic systems^1^,^3^. For example, suppose that the Hamiltonian of some Hubbard-like model contains the nearest-neighbor tunnelling term such as (1). One can argue then that some derivative of the nearest-neighbor correlation function with respect to the tunnelling parameter *J* should be divergent if the change of *J* induces a quantum phase transition. Such a statement should be correct regardless of the specific form of the interaction part of the Hamiltonian, which can contain other than on-site terms. This observation should be useful in both theoretical and experimental studies of the location of the critical point in ubiquitous Hubbard-like models.

## Methods

We use the Directed Worm Algorithm from the ALPS software package^37^,^38^. To evaluate thermodynamical averages, the algorithm samples the space of “worldlines” allowing for the change of the total number of particles. To efficiently evaluate the canonical ensemble averages, the chemical potential is adjusted to yield a unit density in the grand canonical ensemble, thereby maximizing the total number of samples corresponding to the desired average filling factor. In the end, only the “worldlines” with the number of atoms equal to the number of lattice sites are averaged to yield the results presented in Figs 1, 2, 3 and 4.

## Additional Information

**How to cite this article**: Łącki, M. *et al*. Locating the quantum critical point of the Bose-Hubbard model through singularities of simple observables. *Sci. Rep.* **6**, 38340; doi: 10.1038/srep38340 (2016).

**Publisher's note:** Springer Nature remains neutral with regard to jurisdictional claims in published maps and institutional affiliations.
